# Artificial Intelligence, the ChatGPT Large Language Model: Assessing the Accuracy of Responses to the Gynaecological Endoscopic Surgical Education and Assessment (GESEA) Level 1-2 knowledge tests

**DOI:** 10.52054/FVVO.16.4.052

**Published:** 2024-12-27

**Authors:** M Pavone, L Palmieri, N Bizzarri, A Rosati, F Campolo, C Innocenzi, C Taliento, S Restaino, U Catena, G Vizzielli, C Akladios, M.M. Ianieri, J Marescaux, R Campo, F Fanfani, G Scambia

**Affiliations:** UOC Ginecologia Oncologica, Dipartimento di Scienze per la salute della Donna e del Bambino e di Sanità Pubblica, Fondazione Policlinico Universitario A. Gemelli, IRCCS, 00168, Rome, Italy; IHU Strasbourg, Institute of Image-Guided Surgery, 67000 Strasbourg, France; IRCAD, Research Institute against Digestive Cancer (IRCAD) France, 67000 Strasbourg, France; ICube, Laboratory of Engineering, Computer Science and Imaging, Department of Robotics, Imaging, Teledetection and Healthcare Technologies, University of Strasbourg, CNRS, UMR 7357, Strasbourg, France; Department of Obstetrics and Gynecology, University Hospital Ferrara, 44121 Ferrara, Italy; Department of Obstetrics and Gynaecology, University Hospitals Leuven, 3000 Leuven, Belgium; Department of Medical Area (DAME), University of Udine, Clinic of Obstetrics and Gynecology, “Santa Maria Della Misericordia” University Hospital, Azienda Sanitaria Universitaria Friuli Centrale, 33100 Udine, Italy; University Hospitals of Strasbourg, Department of Gynecologic Surgery, 67091 Strasbourg, France; Gynecology and Breast Care Center, Mater Olbia Hospital, Olbia, Italy; Life Expert Centre, Schipvaartstraat 4, 3000 Leuven, Belgium

**Keywords:** ChatGPT, Artificial intelligence, GESEA, laparoscopy, hysteroscopy, digital surgery

## Abstract

**Background:**

In 2022, OpenAI launched ChatGPT 3.5, which is now widely used in medical education, training, and research. Despite its valuable use for the generation of information, concerns persist about its authenticity and accuracy. Its undisclosed information source and outdated dataset pose risks of misinformation. Although it is widely used, AI-generated text inaccuracies raise doubts about its reliability. The ethical use of such technologies is crucial to uphold scientific accuracy in research.

**Objective:**

This study aimed to assess the accuracy of ChatGPT in doing GESEA tests 1 and 2.

**Materials and Methods:**

The 100 multiple-choice theoretical questions from GESEA certifications 1 and 2 were presented to ChatGPT, requesting the selection of the correct answer along with an explanation. Expert gynaecologists evaluated and graded the explanations for accuracy.

**Main outcome measures:**

ChatGPT showed a 59% accuracy in responses, with 64% providing comprehensive explanations. It performed better in GESEA Level 1 (64% accuracy) than in GESEA Level 2 (54% accuracy) questions.

**Conclusion:**

ChatGPT is a versatile tool in medicine and research, offering knowledge, information, and promoting evidence-based practice. Despite its widespread use, its accuracy has not been validated yet. This study found a 59% correct response rate, highlighting the need for accuracy validation and ethical use considerations. Future research should investigate ChatGPT’s truthfulness in subspecialty fields such as gynaecologic oncology and compare different versions of chatbot for continuous improvement.

**What is new?:**

Artificial intelligence (AI) has a great potential in scientific research. However, the validity of outputs remains unverified. This study aims to evaluate the accuracy of responses generated by ChatGPT to enhance the critical use of this tool.

## Introduction

The integration of artificial intelligence (AI) in healthcare has seen a substantial rise recently, marked by a diverse array of applications and technologies which could enhance patient care and support evidence-based medicine. Healthcare providers and systems frequently use such technologies for both patient management and research purposes. In surgery, the first steps in this direction are made through the integration of computer vision and deep learning algorithms, which guide surgical processes by potentially reducing the risk of complications or facilitating the workflow. Additionally, augmented reality and applications of the metaverse in the operating room could allow for better tailoring of surgical procedures (Madani et al., 2024). Although the adoption of new technologies in surgery is increasing, there is a scientific need to demonstrate that they are able to improve clinical outcomes for patients. In 2022, OpenAI launched the ChatGPT chatbot, making it accessible to the public via a free online platform open to all registered users. ChatGPT is trained on a vast dataset, which encompasses a wide spectrum of topics, including the medical literature (Seth et al., 2023). It delivers articulate, engaging, and clear responses, which appear well informed when addressed with questions (Gupta et al., 2023b). There is an ongoing debate about the potential of ChatGPT to revolutionise various academic disciplines, and its practical utility to control the reliability and accuracy of its information (Goglia et al., 2024). Recent research has demonstrated that ChatGPT can take and pass tests such as the United States Medical Licensing Examination (USMLE) and has proven effective in answering queries related to the prevention of cardiovascular diseases and providing accurate responses to common questions about cirrhosis, hepatocellular, and cervical cancers (Hermann et al., 2023; Oh et al., 2023; Gilson et al., 2023). To date, surgical speciality societies have developed training curricula, which include the acquisition and certification of theoretical and practical knowledge using simulators, ensuring that novice surgeons undergo an ethically approved ex vivo training before leading operations (Chen et al., 2020). The European Society for Gynaecological Endoscopy (ESGE) has established the Gynaecological Endoscopic Surgical Education and Assessment (GESEA), a comprehensive training initiative which revolves around theoretical knowledge, practical skills, and the assessment of the reached level of competency (Campo et al., 2016). The first step involves obtaining the GESEA Bachelor Certificate (GESEA 1), affirming a grasp of general endoscopic knowledge and the acquisition of fundamental endoscopic psychomotor skills. The subsequent stage entails the successful completion of the GESEA MIGS (Minimally Invasive Gynaecological Surgeon-GESEA 2) exam, leading to the conferment of the GESEA MIGS Certificate. This certification attests that the trainee has not only mastered advanced knowledge and psychomotor skills, but also possesses the capability to autonomously perform standard procedures in gynaecology (Campo et al., 2016). Considering the widespread use of these new AI tools in scientific research, it is crucial to determine the response reliability of these systems, which have the potential to influence clinical decision- making and patient care when used in medical settings. In this study, we aimed to examine the accuracy of ChatGPT’s responses to the GESEA knowledge tests 1 and 2.

## Materials and Methods

### Large language model

ChatGPT (OpenAI, San Francisco, CA, United States) is a large language model trained on an extensive dataset sourced from various channels, including online platforms, literature, and scholarly articles until year 2021. When presented with queries, ChatGPT demonstrates the ability to deliver well crafted, conversational, and easily digestible responses. Developers used reinforcement learning through human feedback to refine the model’s capacity to interpret a broad spectrum of commands and written directives calibrated based on human preferences as positive reinforcement. Additionally, the model underwent training to align with user objectives while mitigating biases and preventing the generation of dangerous or detrimental responses. The origin of the dataset used to train ChatGPT remains undisclosed.

### GESEA questions

The GESEA Educational Programme operates within a structured framework comprising three proficiency levels, each designed to build upon the foundations laid by the preceding one (Campo et al., 2016). Before advancing to the subsequent level, participants must fulfil the specific criteria set for each tier.

At the initial stage of the programme, the focus is entirely on equipping young surgeons with the essential knowledge and skills necessary for beginning their training within the operating room environment. This foundational level covers a comprehensive range of topics, including laparoscopic suturing techniques, anatomical perspectives relevant to laparoscopy, various methods of entry into the abdomen, exposure surgical techniques, the application of energy, fundamental principles of laparoscopy, as well as the identification and management of associated complications. Additionally, participants delve into the principles of hysteroscopy, exploring both its procedural aspects and the potential complications which may arise.

Upon successful completion of the first level, participants progress to the second tier of the GESEA Educational Programme, where they are primed for undertaking basic endoscopic procedures within the setting of the operating theatre. This intermediate level delves deeper into specific surgical techniques and procedures, including total laparoscopic hysterectomy, myomectomy, interventions for chronic pelvic pain and anterior ligamentopexy, procedures related to pelvic inflammatory disease (PID) and tubal surgery, management of ovarian/adnexal tumours through laparoscopic approaches, emergency laparoscopy, as well as the surgical management of endometriosis for gynaecological surgeons.

### Response generation

The first and second certification levels encompass both theoretical and practical components. The 50 theoretical questions administered during GESEA Level 1 certifications, as well as the 50 questions for GESEA Level 2, were used as prompts for ChatGPT. For each of the 100 multiple-choice questions (with 5 options), ChatGPT was queried in a new chatbox to select the correct answer (only one) and provide explanations for the chosen response. The accuracy of the multiple- choice answers was objectively evaluated based on the correct answers provided in the official GESEA certification database. Meanwhile, the assessment of ChatGPT’s explanations for these answers was conducted by two certified gynaecological surgeons, N.B. and A.R., who are currently practicing and have undergone specialised training. These surgeons were responsible for determining the correctness of the explanations based on information available up to 2021. Each response was graded according to the following criteria: 1. Comprehensive: Indicates accuracy and comprehensiveness, with no additional information required. 2. Correct but lacking in detail: The information provided is accurate but incomplete in some areas; an expert gynaecologist would need to add information to make the explanation comprehensive. 3. Partially correct and partially incorrect: The explanation contains both correct and incorrect content. 4. Completely inaccurate. A third gynaecological surgeon (M.M.I.) with specialised training resolved any discrepancies in assessment. Consistency among evaluators was assessed on the basis of their training and ensured by their GESEA2 certification. To reduce possible bias in the scoring the evaluators were asked to revise twice in different time the ChatGPT evaluations.

### Statistical analysis

The proportions of responses conducive to learning each grade were calculated. Qualitative variables were summarised using absolute and percentage frequency tables. Groups were compared using the Fisher’s exact test or the χ2 test for categorical variables, as deemed appropriate. A p-value < 0.050 was considered statistically significant. All statistical analyses were performed using SPSS version 29.0.1.0 (IBM, Armonk, New York, United States).

## Results

ChatGPT was prompted with 100 multiple-choice questions related to gynaecological endoscopy, for which it provided both the correct answer and an explanation. Fifty-nine percent of responses provided were accurate in selecting the correct answer (see Supplementary Material Appendix 1). Among the explanations provided for the given responses, 64% were considered comprehensive (grade 1), 6% were classified as correct but lacking in details (grade 2), 20% were partially correct and partially incorrect, and 10% were completely inaccurate (grade 4) (Table I). When analysing the data by subgroups, the percentage of accurate responses was 64% for GESEA Level 1 and 54% for GESEA Level 2 (p=0.4). Tables II and III present the specific results categorised per certification level and topic macro-area. Among the explanations provided for the given responses, 70% and 58% (p=0.28) were respectively deemed comprehensive (grade 1) for GESEA 1 and GESEA Level 2. Six percent were classified as correct but lacking in details (grade 2) for both levels (p= 0.58. Additionally, 18% and 22% were partially correct and partially incorrect (p=0.10), whereas 6% and 14% (p= 0.80) were completely inaccurate (grade 4) for GESEA Level 1 and for GESEA Level 2, respectively (Table IV).

**Table I:** General results: total number of correct answers to the multiple-choice questions provided by ChatGPT; Percentage distribution based on expert grading evaluations of the explanations.

Multiple choice questions: Total of correct answers	59 (59%)
Explanation of the given answer: Grading	
1. Comprehensive	64 (64%)
2.Correct but lacking in details	6 (6%)
3.Partially correct and partially incorrect	20 (20%)
4.Completely inaccurate	10 (10%)

**Table II:** Results for GESEA 1 knowledge test. In the table the number of questions by topic (%); The rate of correct answer given by chatGPT (%); the percentage in explanation accuracy as evaluated by experts (%) according to the grading (1. Comprehensive; 2. Correct but lacking in detail; 3. Partially correct and partially incorrect; 4. Completely inaccurate).

TOPIC	Total Number	Correct Answers	Explanation Accuracy
GESEA knowledge test 1	50 (100%)	32 (64%)	1: 35 (70%) 2: 3 (6%) 3: 9 (18%) 4: 3 (6%)
Laparoscopic suturing techniques	7 (14%)	3 (42.8%)	1: 4 (57.1%) 2: 0 (0%) 3: 3 (42.8%) 4: 0 (0%)
Anatomy from a laparoscopic standpoint	8 (16%)	4 (50%)	1: 6 (75%) 2: 2 (25%) 3: 0 (0%) 4: 0 (0%)
Ways of entry	8 (16%)	6 (75%)	1: 6 (75%) 2: 0 (0%) 3: 2 (25%) 4: 0 (0%)
Exposure techniques	2 (4%)	2 (100%)	1: 2 (100%) 2: 0 (0%) 3: 0 (0%) 4: 0 (0%)
Use of energy	2 (4%)	1 (50%)	1: 1 (50%) 2: 0 (0%) 3: 1 (50%) 4: 0 (0%)
Understanding laparoscopy and basic rules	2 (4%)	0 (0%)	1: 0 (0%) 2: 0 (0%) 3: 1 (50%) 4: 1 (50%)
Complications	8 (16%)	5 (62.5%)	1: 5 (62.5%) 2: 1 (12.5%) 3: 1 (12.5%) 4: 1 (12.5%)
Principles of hysteroscopy	6 (12%)	5 (83%)	1: 5 (83.3%) 2: 0 (0%) 3: 0 (0%) 4: 1 (16.6%)
Hysteroscopy complications and management	7 (14%)	6 (85.7%)	1: 6 (85.7%) 2: 0 (0%) 3: 1 (14.2%) 4: 0 (0%)

**Table III:** Results for GESEA 2 knowledge test. In the table the number of questions by topic (%); The rate of correct answer given by chatGPT (%); the percentage in explanation accuracy as evaluated by experts (%) according to the grading (1. Comprehensive; 2. Correct but lacking in detail; 3. Partially correct and partially incorrect; 4. Completely inaccurate).

TOPIC	Total Number	Right Answers	Explanation Accuracy
GESEA knowledge test 2	50	27 (54%)	1: 29 (58%) 2: 3 (6%) 3: 11 (22%) 4: 7 (14%)
Total of laparoscopic hysterectomies	3 (6%)	2 (66.6%)	1: 2 (66.6%) 2: 0 (0%) 3: 0 (0%) 4: 1 (33.3%)
Myomectomy	2 (4%)	1 (50%)	1: 1 (50%) 2: 0 (0%) 3: 0 (0%) 4: 1 (50%)
Chronic pelvic pain and anterior ligamentopexy	3 (6%)	2 (66.6%)	1: 2 (66.6%) 2: 1 (33.3%) 3: 0 (0%) 4: 0 (0%)
PID & Tubal surgery	8 (16%)	5 (62.5%)	1: 2 (25%) 2: 2 (25%) 3: 2 (25%) 4: 2 (25%)
Ovarian/Adnexal Tumours	3 (6%)	0 (0%)	1: 2 (75%) 2: 0 (0%) 3: 1 (25%) 4: 0 (0%)
Laparoscopy in emergency	2 (4%)	2 (100%)	1: 2 (100%) 2: 0 (0%) 3: 0 (0%) 4: 0 (0%)
Endometriosis for the gynaecological surgeon	6 (12%)	6 (100%)	1: 6 (100%) 2: 0 (0%) 3: 0 (0%) 4: 0 (0%)
Hysteroscopic procedures	13 (26%)	8 (61.5%)	1: 8 (61.5%) 2: 4 (30.7%) 3: 0 (0%) 4: 1 (7.6%)
Ways of entry	4 (8%)	2 (50%)	1: 2 (50%) 2: 0 (0%) 3: 2 (50%) 4: 0 (0%)
General laparoscopy	6 (12%)	5 (83.3%)	1: 2 (33.3%) 2: 0 (0%) 3: 2 (33.3%) 4: 2 (33.3%)

**Table IV:** GESEA 1-2 knowledge test results comparison in terms of explanation accuracy according to the grading (1. Comprehensive; 2. Correct but lacking in detail; 3. Partially correct and partially incorrect; 4. Completely inaccurate).

	GESEA knowledge test 1	GESEA knowledge test 2	p value
Correct answers	32	27	0.4
Explaination accuracy			
1	35	29	0.28
2	3	3	0.58
3	9	11	0.10
4	3	7	0.80

## Discussion

From the findings of this study, ChatGPT demonstrated a 59% accuracy rate in answering questions, with a comprehensive explanation provided in 64% of cases. Subgroup analysis revealed that the large language model was more proficient in answering GESEA Level 1 questions (64% of correct responses), as compared to GESEA Level 2 questions (54% of correct responses). Regarding specific topics, the system showed a complete lack of preparedness for questions related to “Understanding Laparoscopy and Basic Rules” and “Ovarian and Adnexal Tumours”.

In 2022, the ChatGPT chatbot was made freely available online in its 3.5 version by the company OpenAI (San Francisco, CA, United States). The system’s ability to generate information when freely queried has revolutionised numerous sectors of society (Wang et al., 2023). In the medical field, this large language model is widely used for educational and training purposes, whereas it plays an increasingly significant role in information generation for article writing and bibliographic research in the scientific setting (Beaulieu-Jones et al., 2023). If used correctly, these new technologies represent an unprecedented opportunity for aiding scientific research. However, to date, the source of the generated information is unknown, and officially, the dataset is only updated until 2021, posing a risk of outdated information (Lim et al., 2023).

Some authors already use ChatGPT or other language models for the actual drafting of articles, and certain publishers allow these systems to be listed as co-authors (Levin et al., 2023). However, there have been recent cases where texts generated by means of large language models, without human review, contained circumstantial phrases clearly generated by artificial intelligence, raising doubts about the scientific accuracy of the entire information presented (Zhang et al., 2024;). Additionally, although the use of such systems may facilitate article writing, questions arise regarding the truthfulness of the information and the level of accuracy they can achieve (Wójcik et al., 2023).

There is a need to objectively assess the system’s readiness to validate its potential use in scientific settings (Thirunavukarasu et al., 2023). With this aim, several authors have queried ChatGPT with questions taken from official medical certifications to assess its preparedness and evaluate the level of proficiency that artificial intelligence can achieve with similar results obtained in this study. Takagi et al. reported an accuracy of 50.8% in responding to questions from the Japanese Medical Licensing Examination (JMLE) (Takagi et al., 2023). When evaluating performance across four datasets of the United States Medical Licensing Examination (USMLE) — AMBOSS-Step 1, AMBOSS-Step 2, NBME-Free-Step 1, and NBME-Free-Step 2 — ChatGPT achieved accuracies of 44% (44/100), 42% (42/100), 64.4% (56/87), and 57.8% (59/102), respectively (Gilson et al., 2023). In the field of specialised surgeries, ChatGPT answered a total of 242 questions with an accuracy of 54.96% in the theoretical certification required for plastic surgery residents (Gupta et al., 2023a). Meanwhile, in a study by Lum (2023), 400 out of 3,840 publicly available questions based on the Orthopaedic In- Training Examination were presented to ChatGPT and compared with the mean score of residents who took the test over a 5-year period. ChatGPT selected the correct answer in 47% (97 of 207) of the time, and in 53% (110 of 207) of the time it answered incorrectly, placing it at the 40th percentile as compared to residents’ responses.

The validity of the information provided was also assessed by evaluating the system’s ability to respond to questions posed by patients in the field of bariatric surgery (Samaan et al., 2023). In gynaecology, to date, only three studies have been published on this topic. In the first study, the accuracy of ChatGPT in responding to commonly asked questions regarding cervical cancer prevention, diagnosis, treatment, and survivorship/ quality of life (QOL) was quantified. ChatGPT provided correct and comprehensive answers in 53.1% of cases (Hermann et al., 2023). In the second study, a bibliometric analysis demonstrated that there are currently no studies incorporating ChatGPT into the drafting of scientific articles in the field of gynaecology (Levin et al., 2023). Finally, Levin et al. (2024) published a study aimed to assess the impact of reviewer experience on distinguishing between human-written and ChatGPT-written abstracts. Thirty reviewers evaluated 10 human-written and 10 ChatGPT- generated abstracts, resulting in 600 evaluations. Human-written abstracts were identified correctly at a higher rate (53.7%), as compared to ChatGPT- generated ones (46.3%). Senior reviewers demonstrated a significantly higher correct identification rate (60%) than junior reviewers and residents (45% each), and familiarity with artificial intelligence was associated with improved identification accuracy. Additionally, reviewer publication experience positively correlated with the correct identification rate, underscoring the importance of experience and familiarity with AI in accurately discerning between human and AI- generated content (Levin et al., 2024).

Recently, Goglia et al. (2024) published the first systematic review covering all areas of the application of ChatGPT in abdominopelvic surgery. The study shows how, in addition to educational and training purposes, large language models (LLMs) are increasingly integrated into clinical practice, serving as aids in the management of complex cases and emergencies, in multidisciplinary tumour boards to assist in difficult decisions, and in the drafting of operative reports.

This is the first study to investigate the validity of information provided by ChatGPT in the field of gynaecological surgery. The results reveal an accuracy rate of 59%, consistent with previous studies published in the literature, in which the large language model was queried with questions from official medical certifications (7, 18-20). The system’s ability to provide comprehensive explanations (64%), even when the response is incorrect, suggests a potential for improvement. Additionally, the total inaccuracy in responses within specific subgroups, such as oncological gynaecology, cannot be statistically considered due to the limited number of questions.

A limitation of this study lies in the potential bias in response generation, caused by the ambiguous formulation of questions interpreted by the large language model, which may have led to erroneous responses. This bias degree could represent the major limitation of the study highlighting the potential for using chatbots to create unambiguous questions for certification purposes. Additionally, the lack of knowledge about the dataset from which Chat GPT derives its information can lead to issues with reproducibility. This not only represents a limitation of the study but also focuses the light on a general lack of reliability.

As of now, newer versions of the chatbot are available for purchase. ChatGPT 4 has been declared capable of providing more accurate information in technical and specialised fields, including healthcare, and has integrated the capability for image input analysis (Almazyad et al., 2023)

The rapid integration of ChatGPT into everyday life, coupled with ongoing improvements in its capabilities, suggests that it will become increasingly integrated across all fields (Srinivasan, unpublished data). In surgery, the continued development of new robotic platforms (Pavone et al., 2024a) that excel in interfacing with artificial intelligence systems , image-guided surgery , and augmented reality opens the door to potential integrations with large language models (Pavone et al., 2024b). It could well lead to direct interactions during surgery between surgeons and machines, which would not only assist in mechanical procedures but also provide theoretical comparisons influencing decision-making. In the countless future scenarios, it remains critical to demonstrate the validity and accuracy of the information that such systems generate, with an ethical focus on developing technologies to enhance clinical practice and patient care.

## Conclusion

Based on the results of this study, ChatGPT demonstrated a correct response rate of 59% and provided comprehensive responses in 64% of cases. As ChatGPT becomes increasingly integrated into medical practice and research, it is crucial to control the accuracy and reliability of the information provided. Ethical considerations must be taken into account when using artificial intelligence tools such as ChatGPT in medical settings, as they have the potential to influence clinical decision-making and patient care. Future studies should focus on investigating the accuracy of information provided by ChatGPT, particularly in specialised fields such as oncological gynaecology. They should also focus on comparing the accuracy of given outputs of different versions of the system to ensure continuous improvement of emerging technologies in scientific research.
