# Supplementary material for: Artificial Intelligence, the ChatGPT Large Language Model: Assessing the Accuracy of Responses to the Gynaecological Endoscopic Surgical Education and Assessment (GESEA) Level 1-2 knowledge tests
Source: Facts Views Vis Obgyn. 2024 Dec 27;16(4):449–56. doi: 10.52054/FVVO.16.4.052 (PMC11819790; doi:10.52054/FVVO.16.4.052)
Supplement: Supplementary file 1 [file FVVinObGyn-16-449-app1.pdf]

Appendix 1: Below is an example of chatbot prompting, along with the generated output and the expert's evaluation score.

n	GESEA TEST1					
	**QUESTION**	**REPLY**	**CORRECT**	**EXPLAINATION**	**Evaluator 1**	**Evaluator 2**
1	SELECT THE RIGHT ANSWER AND GIVE AN EXPLAINATION:<br>During diagnostic hysteroscopy, the first instrument to be inserted (into the cervical canal) should be:<br>□ Uterine probe<br>□ Hegar's dilator<br>□ Hysteroscope<br>□ Grasping forceps<br>□ Swab	Hysteroscope	YES	During diagnostic hysteroscopy, the first instrument to be inserted into the cervical canal is the hysteroscope. This is because the hysteroscope allows visualization of the uterine cavity, which is essential for diagnosing any abnormalities or conditions within the uterus. Once the hysteroscope is inserted, other instruments may be used as needed for further evaluation or treatment.	1	1
2	SELECT THE RIGHT ANSWER AND GIVE AN EXPLAINATION:<br><br>If you use a 5mm instrument in a 10mm port, you can provoke the following situation:<br>□ Higher risk of patient injury<br>□ Gas loss<br>□ Loss of precision in movements<br>□ Needle loss	Loss of precision in movements	YES	Using a 5mm instrument in a 10mm port can result in a loss of precision in movements. When the instrument is smaller than the port, there is more room for the instrument to move around within the port, which can lead to less control and accuracy during surgical procedures. This can potentially increase the risk of errors or complications during the procedure.	1	1

	□ Higher risk of port slipping					
3	SELECT THE RIGHT ANSWER AND GIVE AN EXPLAINATION:<br><br>In an attempt to prevent postoperative surgical incision hernias, studies have shown that the surgeon should close the abdominal wall fascia:<br>□ In all patients<br>□ In patients with incisions of 3mm or larger<br>□ In patients with incisions of 5mm or larger<br>□ In patients with incisions of 7mm or larger<br>□ In patients with incisions of 10mm or larger	In patients with incisions of 10mm or larger	YES	Studies have shown that closing the abdominal wall fascia is important for preventing postoperative surgical incision hernias, especially in patients with incisions of 10mm or larger. Closing the fascia helps to provide additional support to the abdominal wall, reducing the risk of hernias developing at the surgical site. For smaller incisions, the fascia may not need to be closed as rigorously, but for larger incisions, it is generally recommended to close the fascia to prevent complications.	1	1

4	SELECT THE RIGHT ANSWER AND GIVE AN EXPLAINATION:<br><br>What is the more frequent location of major vessel injury during umbilical trocar<br>insertion?<br>□ Bifurcation of aorta<br>□ Right common iliac artery<br>□ Right common iliac vein<br>□ Left common iliac artery<br>□ Left common iliac vein	Right common iliac artery	WRONG	During umbilical trocar insertion, the more frequent location of major vessel injury is the right common iliac artery. This is because of its proximity to the insertion site and the trajectory of the trocar. Careful insertion technique and awareness of the anatomical landmarks can help minimize the risk of such injuries.	3	3
